# Changes in the Mean and Variance of the Numbers of Medical Visits for Allergic Diseases before and during the COVID-19 Pandemic in Korea

**DOI:** 10.3390/jcm11154266

**Published:** 2022-07-22

**Authors:** Hyo Geun Choi, Joo-Hee Kim, Yong-Hwi An, Min Woo Park, Jee Hye Wee

**Affiliations:** 1 Department of Otorhinolaryngology-Head & Neck Surgery, Hallym University Sacred Heart Hospital, Hallym University College of Medicine, Anyang 14068, Korea; pupen@naver.com; 2 Hallym Data Science Laboratory, Hallym University College of Medicine, Anyang 14068, Korea; 3 Division of Pulmonary, Allergy and Critical Care Medicine, Department of Medicine, Hallym University Sacred Heart Hospital, Hallym University College of Medicine, Anyang 14068, Korea; luxjhee@gmail.com; 4 Department of Otorhinolaryngology-Head & Neck Surgery, Nowon Eulji Medical Center, Eulji University College of Medicine, Seoul 01830, Korea; an0072@hanmail.net; 5 Department of Otorhinolaryngology-Head & Neck Surgery, Kangdong Sacred Heart Hospital, Seoul 05355, Korea; subintern@naver.com

**Keywords:** COVID-19, allergic rhinitis, asthma, atopic dermatitis, allergic conjunctivitis, SARS-CoV-2

## Abstract

The implementation of precautionary measures, such as wearing a mask and social distancing, may have affected allergic diseases during the coronavirus disease 2019 (COVID-19) pandemic. This study aimed to compare the numbers of medical visits for allergic diseases before and during the COVID-19 pandemic. Data were obtained from the Korean National Health Insurance claims database. Monthly numbers of patients for four allergic diseases, i.e., allergic rhinitis (AR), asthma, atopic dermatitis (AD), and allergic conjunctivitis (AC), were evaluated using ICD-10 codes and compared between the ‘before COVID-19’ period from January 2018 to February 2020, and the ‘during COVID-19’ period from March 2020 to June 2021, since the first COVID-19 patient was detected on 20 January 2020, in Korea. Subgroup analyses were performed according to age and sex. The mean numbers of medical visits for AR and asthma were significantly greater before COVID-19 than those during COVID-19 (both *p* < 0.001). The variance in the number of medical visits for asthma decreased during the COVID-19 pandemic. However, the mean number of medical visits for AD increased slightly during COVID-19 and that for AC did not change before and during the COVID-19 pandemic. In subgroup analyses, the results showed a similar pattern to that of the total number of participants, regardless of age and sex. In conclusion, medical visits for AR and asthma significantly decreased during the COVID-19 pandemic, regardless of age and sex.

## 1. Introduction

Guidelines regarding the management of allergic diseases have recently been published to maintain high standards in clinics and ensure necessary safety during the coronavirus disease 2019 (COVID-19) pandemic [1,2]. Furthermore, the implementation of strict measures to combat the spread of severe acute respiratory syndrome coronavirus 2 (SARS-CoV-2) has had a significant impact on individual behaviors, although the situation varies across countries.

Studies have reported conflicting results regarding the effect of allergic diseases on the course of COVID-19. The prevalence of allergic diseases varies considerably among patients with COVID-19 in different countries or regions. A systematic review and meta-analysis revealed that preexisting allergic diseases did not deteriorate the course of COVID-19 and tended to be significantly associated with a decreased mortality risk of COVID-19 [3]. In contrast, in a Korean nationwide cohort study, the presence of allergic rhinitis (AR) and asthma conferred a greater risk of SARS-CoV-2 infection and serious negative clinical outcomes of COVID-19 [4]. This may be because of racial differences among patients or differences in the medical infrastructure among countries. Increased fear of COVID-19 may result in decreased utilization of healthcare for underlying diseases.

Few studies have assessed the change in the number of patients treated for allergic diseases before and during the COVID-19 pandemic. In a recent study using data from the Korean National Health and Nutrition Examination Survey (KNHANES) 2008–2017, the 10-year trend of asthma prevalence remained low and stable, AR prevalence increased or remained stable, and atopic dermatitis (AD) prevalence decreased among infants and preschool children and increased in school-age children and elderly individuals; however, the observed period preceded the COVID-19 pandemic [5]. Although a recent study reported that the spread of COVID-19 significantly reduced the incidence of other respiratory diseases, including AR, sinusitis, acute upper respiratory infections, and bronchopneumonia [6], there were no reports on the incidence of asthma, AD, and allergic conjunctivitis (AC).

The aim of this study was to investigate the change in the mean numbers of medical visits for allergic diseases between the non-pandemic and COVID-19 pandemic periods in the Korean population. In addition, we evaluated the variance or seasonality in the number of medical visits for allergic diseases during the COVID-19 pandemic.

## 2. Materials and Methods

### 2.1. Study Population and Data Collection

This study included the entire Korean population (~50 million). This was possible because Korea has a single mandatory national health insurance system that covers almost the entire population, and access to medical care, including tertiary care, is easy. Thus, we gathered the data of all Koreans from primary clinics to tertiary hospitals. Data were obtained from the Korean National Health Insurance claims database. This study was approved by the Institutional Review Board (IRB) of Hallym University (IRB no. 2021-11-004). The requirement for written informed consent was waived because of the retrospective design of the study.

We evaluated the monthly number of patients treated for allergic diseases between January 2018 and June 2021. As the first patient with COVID-19 was reported on 20 January 2020, in Korea, and disease prevention and control began in March 2020, the period ‘before COVID-19’ was defined until 29 February 2020, and ‘during COVID-19′ was defined from 1 March 2020. The diagnosis of the four allergic diseases was based on the following ICD-10 codes: AR (J301, J302, J303, and J304), asthma (J45 and J46), AD (L20), and AC (H101). In Korea, antihistamines cannot be purchased without a prescription; therefore, patients must visit a clinic or hospital for the treatment of allergic diseases. The monthly number of patients treated for allergic diseases was calculated without duplication as we had the medical records of all hospitals and clinics, and all patients were identified by their unique resident registration number.

### 2.2. Statistical Analysis

The mean number of medical visits for each allergic disease before and during the COVID-19 pandemic was compared using the Mann–Whitney U test. The variances in the number of medical visits for each allergic disease before and during the COVID-19 pandemic were compared using the Levene’s test [7]. For subgroup analyses, we classified the participants according to sex (male and female) and age (0–19 years, 20–59 years, and 60+ years). Furthermore, in relation to pediatric patients between the age of 0 and 19, we divided them by age into subgroups of 5 year intervals (0–4 years, 5–9 years, 10–14 years, and 15–19 years).

Two-tailed analyses were conducted, and statistical significance was defined as *p* < 0.05. Statistical analyses were performed using the SPSS version 22.0 (IBM, Armonk, NY, USA).

## 3. Results

**Figure 1:**
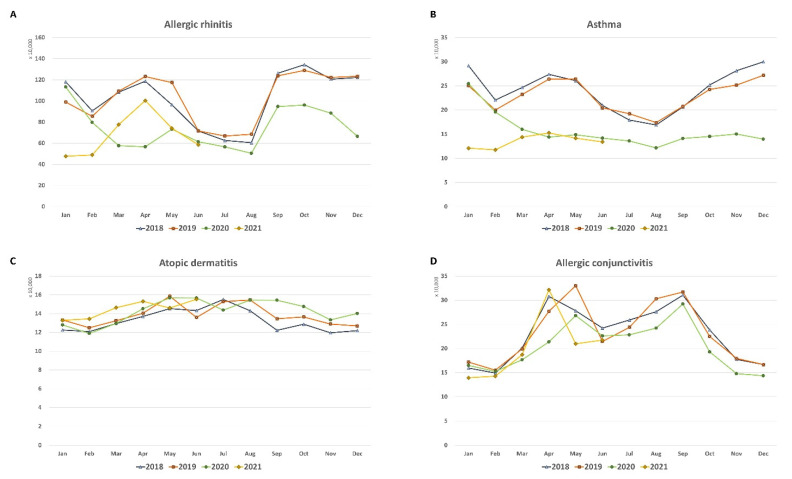
Monthly numbers of medical visits for allergic diseases (January 2018–June 2021): (**A**) Allergic rhinitis; (**B**) asthma; (**C**) atopic dermatitis; (**D**) allergic conjunctivitis.

Table 1 shows the differences in the monthly numbers of medical visits for allergic diseases before and during the COVID-19 pandemic. The mean numbers of medical visits for AR and asthma were significantly greater before COVID-19 than that during COVID-19 (both *p* < 0.001). The variance in the number of medical visits for asthma was significantly greater before COVID-19 than that during COVID-19 (*p* = 0.024). Although the monthly number of patients treated for AR continued to be seasonal, the seasonality of asthma decreased during the COVID-19 pandemic (Figure 1). However, the mean medical visits for AD slightly increased during the COVID-19 pandemic (*p* = 0.003). The mean medical visits for AC did not differ significantly before and during the COVID-19 pandemic (*p* = 0.365). The variance in the monthly number of patients treated for AD (*p* = 0.158) and AC (*p* = 0.619) did not differ before and during the COVID-19 pandemic.

The results of subgroup analyses according to sex (Table 2) and age (Table 3) were similar to those of the total number of participants. In men and women, the mean numbers of medical visits for AR and asthma were significantly greater before COVID-19 than those during COVID-19 (all *p* < 0.001, Table 2). The variance in the number of medical visits for asthma was significantly greater before COVID-19 than that during COVID-19 (*p* = 0.024 in men and *p* = 0.026 in women). In all subgroups according to age, higher monthly numbers of patients treated for AR and asthma were observed before COVID-19 than those during COVID-19 (all *p* < 0.05, Table 3). The decreases in the monthly numbers of patients treated for AR and asthma tended to be more pronounced in women and the age subgroup of 20–59 years. However, the mean number of medical visits for AD slightly increased during the COVID-19 pandemic, and the mean number of medical visits for AC did not differ significantly in men and in women (Table 2) and before and during the COVID-19 pandemic. The mean and variance in the monthly numbers of patients treated for AD and AC demonstrated no significant difference before and during the COVID-19 pandemic in the age subgroup of 0–19 years (all *p* > 0.05, Table 3).

In the additional subgroup analyses of pediatric patients at 5-year intervals (Table 4), the results were similar to those of all pediatric participants. The mean numbers of medical visits for AR and asthma was significantly decreased during COVID-19 (all *p* < 0.05). Although the mean monthly number of children treated for AC significantly decreased only in the subgroup of 0–4 years (*p* = 0.008), in all other subgroups, the mean numbers of medical visits for AD and AC did not change during COVID-19 (all *p* > 0.05).

## 4. Discussion

The findings obtained from this study indicate that the mean numbers of medical visits for AR and asthma decreased, and those for AD and AC were similar before and during the COVID-19 pandemic, regardless of age and sex.

Some possible reasons for the decrease in the mean numbers of medical visits for AR and asthma during the COVID-19 pandemic can be proposed. First, this decrease could be attributed to the relationship between infectious and allergic diseases. Infectious agents have been identified as the causative factors of allergic diseases [8]. Respiratory infections caused by viruses, such as rhinoviruses and respiratory syncytial virus, have been recognized as the dominant causes of symptom exacerbation in patients with asthma [9,10]. When respiratory viruses infect the airway epithelium, type 2 inflammation is promoted by eosinophils, the predominant cellular component of inflammation [11]. Wearing masks and social distancing played crucial roles in preventing the spread of the COVID-19 pandemic. These measures contributed to the decline in the prevalence of other infectious diseases, including respiratory viral infections [12,13]. Second, wearing face masks can reduce atopic allergic responses. A decrease in symptom severity with mask use in patients with AR has been reported [14]. Mechanisms such as physical filtration by face masks and potentially modified physiological responses to allergens by breathing humid and hot air have been proposed. In the present study, though the mean numbers of medical visits for AR and asthma decreased, the mean number of medical visits for AC did not. This can be explained by the fact that face masks potentially lower the burden of inhaled airborne particles, including allergens and air pollutants, while the conjunctiva remains exposed to allergens. Third, improved air quality during the COVID-19 pandemic may have reduced the severity of AR and asthma. Reductions in air pollution levels have been reported in several countries worldwide [15,16,17]. Air pollution has been associated with respiratory diseases such as AR [18] and asthma [19]. However, a definitive causal explanation requires further analysis.

In contrast, the mean medical visits for AD slightly increased during the COVID-19 pandemic. It may be due to the adverse dermatologic effects of precautionary measures, such as wearing a mask and washing hand frequently. Systematic reviews have shown that wearing face masks to protect from COVID-19 can increase adverse facial dermatoses and exacerbate underlying dermatology conditions [20], and frequent hand washing for COVID-19 prevention can cause hand dermatitis, especially in individuals with a history of atopic dermatitis [21]. However, in the pediatric subgroup, the mean and variance in the monthly number of patients treated for AD demonstrated no significant difference before and during the COVID-19 pandemic. In a previous study showing time trends in the prevalence of AD in Korean children under the age of 18, the prevalence of AD has been on decreasing trends since 2008 [22]. Therefore, it is considered mixing effects of decreasing trends of prevalence of AD and adverse dermatologic effects in pediatric population.

The seasonality in the number of medical visits for asthma was attenuated during the COVID-19 pandemic. This finding is consistent with the intended function of facemasks in reducing the burden of inspiratory particles, including air pollutants and aeroallergens. A population-based study in Taiwan showed that seasonal variations in asthma were significantly positively correlated with levels of pollutants in the air, including levels of PM_10_, SO_2_, CO, and NO_2_ [23]. In a randomized, controlled crossover study in China, facemasks filtered 48–75% of ambient air particles that had a diameter between 5.6 nm and 560 nm, and real facemasks attenuated the effects of pollution on respiratory inflammation to a greater extent than sham facemasks [24]. In addition, pollen levels show a seasonal pattern, and wearing facemasks during the pollen season was recommended as an effective nonpharmacological option for patients with pollen allergies [25]. A previous study performed in Spain showed that high pollen levels caused an increase in hospitalizations due to asthma [26]. A prospective questionnaire-based study in Turkey reported that exposure to pollen and allergic symptoms could be reduced in patients with pollen allergies by reducing outdoor activity during the pollen season and wearing a mask [27].

Few studies performed in pediatric populations have shown the effects of lockdown on allergic symptoms. A study including French children with persistent asthma showed fewer exacerbations, better asthma symptom control, and improved lung function after the COVID-19 outbreak [28]. An Italian survey conducted in a pediatric population with AR and/or asthma showed a general trend of clinical improvement and reductions in on-demand and basal therapy during the COVID-19 lockdown [29]. The present study was a population-based study that included participants of all ages, including children and adults, and although there was no lockdown period in Korea, the results were similar to those of previous studies.

This study has some limitations. First, the decrease in the numbers of medical visits for AR and asthma found in the present study may not reflect a reduction in the prevalence of allergic diseases because we only assessed the medical records of hospitals and clinics. Patients may not have visited clinics or hospitals despite needing treatment for allergic diseases. However, in Korea, a nationwide lockdown has never been imposed, and antihistamines cannot be purchased without a prescription. Therefore, patients with allergic diseases in Korea must visit a clinic or hospital for treatment. Furthermore, since the National Health Insurance system covers almost the entire population and is cost-effective, patients with allergic diseases often visit clinics or hospitals even if their symptoms are mild. Second, we could not obtain information regarding patients’ psychological disturbances such as “coronaphobia” [30]. Increased fear of COVID-19 may result in decreased utilization of healthcare for allergic diseases. However, the investigation period was longer than one year to reduce selection bias. Third, information regarding whether wearing masks and social distancing was well implemented by the participants could not be obtained. However, in a survey by the Gallup International Association, Korea reported the highest rates of using medical masks (94%) and hand sanitizers (73%), washing hands frequently (92%), and staying home or less social interaction (85%) among 28 countries, demonstrating strong implementation of precautionary measures [31]. Fourth, data regarding symptom scores and use of medications, such as antihistamines and intranasal corticosteroid spray, could not be obtained. Lastly, we could not measure the levels of pollen and air pollutants. However, it was reported that the mean levels of PM_2.5_, PM_10_, NO_2_, and CO decreased nationwide during social distancing after the outbreak of COVID-19 in Korea [32]. Despite these limitations, the strength of this study is that it is a population-based study with a large number of participants that compared the mean and variance of the numbers of medical visits for allergic diseases before and during the COVID-19 pandemic.

## 5. Conclusions

The diagnosis and treatment of AR and asthma decreased significantly during the COVID-19 pandemic, regardless of age and sex. The results suggest that the widespread use of precautionary procedures, such as wearing a mask and social distancing, contributed to the prevention of COVID-19 and reduced the number of medical visits for respiratory allergic diseases.

## Figures and Tables

**Table 1:** Monthly numbers of patients treated for allergic diseases before and during the COVID-19 pandemic and their differences.

Allergic Diseases	Before COVID-19		During COVID-19		*p*-Values of Difference	
	Mean	SD	Mean	SD	Mean	Variance
Allergic rhinitis	1,024,274.8	239,258.5	693,095.7	176,630.0	<0.001 ^1^	0.400
Asthma	234,469.5	37,564.4	139,927.4	11,694.9	<0.001 ^1^	0.024 ^2^
Atopic dermatitis	134,468.9	11,731.9	145,643.5	9249.4	0.003 ^1^	0.158
Allergic conjunctivitis	225,691.5	59,664.5	209,422.4	54,139.8	0.365	0.619

SD, standard deviation. ^1^ Mann Whitney U test, significance at *p* < 0.05. ^2^ Levene’s test for non-parametric data, significance at *p* < 0.05.

**Table 2:** Monthly numbers of patients treated for allergic diseases before and during the COVID-19 pandemic and the differences in the subgroups divided by sex.

Allergic Diseases	Before COVID-19		During COVID-19		*p*-Values of Difference	
	Mean	SD	Mean	SD	Mean	Variance
**Men**						
Allergic rhinitis	491,945.7	114,174.0	350,631.1	92,822.7	<0.001 ^1^	0.441
Asthma	108,406.9	16,640.8	65,749.5	5444.8	<0.001 ^1^	0.024 ^2^
Atopic dermatitis	67,827.2	5205.8	72,180.6	4250.5	0.010 ^1^	0.231
Allergic conjunctivitis	88,548.7	27,802.9	82,416.4	26,359.6	0.453	0.694
**Women**						
Allergic rhinitis	532,329.1	125,692.6	342,464.6	84,306.7	<0.001 ^1^	0.367
Asthma	126,062.6	21,198.4	74,177.9	6463.7	<0.001 ^1^	0.026 ^2^
Atopic dermatitis	66,641.8	6634.1	73,462.9	5113.2	0.002 ^1^	0.210
Allergic conjunctivitis	137,142.8	32,273.5	127,006.0	28,456.9	0.312	0.482

SD, standard deviation. ^1^ Mann Whitney U test, significance at *p* < 0.05. ^2^ Levene’s test for non-parametric data, significance at *p* < 0.05.

**Table 3:** Monthly numbers of patients treated for allergic diseases before and during the COVID-19 pandemic and the differences in the subgroups divided by age.

Allergic Diseases	Before COVID-19		During COVID-19		*p*-Values of Difference	
	Mean	SD	Mean	SD	Mean	Variance
**Age 0–19 years old**						
Allergic rhinitis	466,299.4	110,618.7	321,090.3	109,387.0	0.001 ^1^	0.697
Asthma	69,732.0	17,899.0	24,172.8	5679.8	<0.001 ^1^	0.024 ^2^
Atopic dermatitis	68,771.1	7007.3	69,566.8	4882.7	0.679	0.133
Allergic conjunctivitis	73,822.6	26,621.0	62,921.3	23,361.6	0.187	0.903
**Age 20–59 years old**						
Allergic rhinitis	410,159.9	111,158.4	259,942.0	72,540.6	<0.001 ^1^	0.281
Asthma	77,006.5	11,790.6	49,716.7	4634.4	<0.001 ^1^	0.033 ^2^
Atopic dermatitis	52,340.8	5485.0	61,514.6	4849.9	<0.001 ^1^	0.367
Allergic conjunctivitis	105,302.8	27,828.3	98,822.4	25,068.3	0.393	0.383
**Age 60+ years old**						
Allergic rhinitis	148,223.2	40,102.8	112,359.7	19,818.1	0.002 ^1^	0.013 ^2^
Asthma	87,873.7	13,088.0	66,115.1	4344.6	<0.001 ^1^	0.112
Atopic dermatitis	13,426.7	983.1	14,645.6	1163.7	0.002 ^1^	0.996
Allergic conjunctivitis	46,614.5	6953.1	47,722.6	7309.5	0.604	0.953

SD, standard deviation. ^1^ Mann Whitney U test, significance at *p* < 0.05. ^2^ Levene’s test for non-parametric data, significance at *p* < 0.05.

**Table 4:** Monthly numbers of pediatric patients treated for allergic diseases before and during the COVID-19 pandemic and the differences in the subgroups divided into five-year intervals.

Allergic Diseases	Before COVID-19		During COVID-19		*p*-Values of Difference	
	Mean	SD	Mean	SD	Mean	Variance
**Age 0–4 years old**						
Allergic rhinitis	190,691.7	32,802.2	129,371.1	47,106.1	<0.001 ^1^	0.619
Asthma	36,364.3	9560.8	10,540.7	3029.2	<0.001 ^1^	0.024 ^2^
Atopic dermatitis	26,972.8	2665	27,599.6	2481.1	0.551	0.229
Allergic conjunctivitis	16,379.4	4534	12,461.6	3075	0.008 ^1^	0.663
**Age 5–9 years old**						
Allergic rhinitis	152,168.9	37,692.8	113,946.6	39,225.4	0.007 ^1^	0.816
Asthma	21,256.7	5652.7	7752.6	1954.8	<0.001 ^1^	0.024 ^2^
Atopic dermatitis	16,351.3	2058.1	16,240.7	1446.1	0.877	0.148
Allergic conjunctivitis	29,798.2	11,726	26,872.6	11,364.6	0.351	0.803
**Age 10–14 years old**						
Allergic rhinitis	72,561.8	25,390.1	47,439.8	20,278	0.001 ^1^	0.878
Asthma	7368.3	2133.4	3286.4	626.3	<0.001 ^1^	0.028 ^2^
Atopic dermatitis	11,391.5	1565.7	12,083.1	1196.1	0.070	0.082
Allergic conjunctivitis	16,263.6	6785	14,201.1	5903.8	0.338	0.746
**Age 15–19 years old**						
Allergic rhinitis	51,311.7	20,819.6	30,881.1	15,460.7	<0.001 ^1^	0.809
Asthma	4823.1	1335.1	2624	435.5	<0.001 ^1^	0.076
Atopic dermatitis	14,096.4	1508.1	13,736.9	1191.6	0.641	0.403
Allergic conjunctivitis	11,403.9	4424.1	9430.5	3560.2	0.126	0.549

SD, standard deviation. ^1^ Mann Whitney U test, significance at *p* < 0.05. ^2^ Levene’s test for non-parametric data, significance at *p* < 0.05.

## Data Availability

Researchers are not legally permitted to release the data. All data are available from the database of the Korea Center for Disease Control and Prevention. The Korea Center for Disease Control and Prevention allows data access, at a cost, to any researcher who promises to follow research ethics. The data used in this study can be downloaded from the website after agreeing to follow research ethics.
